# The effect of metformin on low birth weight girls with precocious puberty: A protocol for systematic review and meta-analysis

**DOI:** 10.1097/MD.0000000000029765

**Published:** 2022-06-30

**Authors:** Zhiheng Lin, Xiaohui Sui, Lijuan Li, Ying Wang, Junde Zhao

**Affiliations:** a Shandong University of Traditional Chinese Medicine, Jinan, Shandong, China; b Changchun University of Science and Technology, Changchun, Jilin, China.

**Keywords:** biguanides, metabolism, LBW-PP girls

## Abstract

In recent years, the role of metformin in girls with precocious puberty (PP) has been increasingly frequently studied. The objective of this present study is to assess the effect of metformin on low birth weight girls with precocious puberty (LBW-PP girls).

**Methods:**

We search the confirmed studies about circulating metformin and PP from the databases of EMBASE, PubMed, and Web of Science. Data were reported as weighted mean difference (WMD) and associated 95% confidence intervals (CIs). Analysis was performed by Review Manager 5.3 and Stata version 12.0.

**Results:**

A total of 205 cases (metformin group n = 102, untreated group n = 103) were included in this study. The meta-analysis of randomized controlled trials (RCTs) suggested that metformin had statistically significant effects on testosterone (*P* = .001), androstenedione (*P* = .022), bone mineral density (BMD; *P* = .151), triglycerides (*P* ≤ .001), body mass index *Z* score (BMI *Z* score; *P* ≤ .001), dehydroepiandrosterone-sulfate (DHEAS; *P* = .053), sex hormone-binding globulin (SHBG; *P* = .049), high-density lipoprotein (HDL) cholesterol (*P* ≤ .001), low-density lipoprotein (LDL) cholesterol (*P* = .021), fat mass (*P* ≤ .001), lean mass (*P* = .025), and fasting insulin (*P* = .002).

**Conclusion:**

This meta-analysis provided evidence of the efficacy of metformin in girls with LBW-PP girls, which proved that metformin could improve metabolism and reduce weight. Metformin had a positive effect on preventing LBW-PP girls from developing into obesity and polycystic ovarian syndrome. In addition, this meta-analysis provided important reference opinions and directions for the treatment of LBW-PP girls.

## 1. Introduction

Female precocious puberty (PP) represents a girl’s development of secondary sexual characteristics before the age of 8.^[1]^ PP is classified into 2 major categories based on the etiology: central PP (gonadotropin-releasing hormone [GnRH] dependent) and peripheral precocious puberty (PPP; GnRH independent).^[1,2,6,7]^ PPP is due to the production of sex steroids from endogenous or exogenous sources.^[2,6,7]^ Precocious development of secondary sexual characteristics is independent of the GnRH pulsatile secretion.^[6,7]^ Some important causes include congenital adrenal hyperplasia, McCune Albright syndrome, gonadal tumors, adrenal tumors, familial male, exogenous exposure to sex steroids, Van Wyk, and Grumbach syndrome.^[3,6,7]^ It is reported that some low birth weight girls with PP (LBW-PP) girls tend to start puberty earlier with too large adrenarcheal hormone profile due to excessive catch-up of growth.^[3,4]^ PP may lead to some physical and psychological changes, for the growth plate is obviously sensitive to the effects of estrogen and the nutritional consequences of PP tend to precede the signs of sexual maturity, which may result in premature fusion of the growth plate, thus leading to short stature in adults.^[5]^ In recent years, the incidence of PP has increased year by year.^[6]^ In addition, low birth weight girls would eventually develop polycystic ovary syndrome (PCOS). In this process, girls at risk for development of an adipose body composition, with early onset of puberty and rapid progression to menarche, exaggerated their weight catch-up in early childhood and showed development of PP.^[8–10]^

Metformin, dimethylbiguanide, is an oral glucose-lowering agent.^[12]^ Metformin seems to function mainly through activation of adenosine monophosphate–activated protein kinase, a conserved regulator of the cellular response to low energy in the liver. This activation is catalyzed by serine-threonine-kinase (LKB1).^[11–14]^ In addition, metformin is widely used in the treatment of tumors, diabetes, and other diseases.^[15,16]^ Recent studies have shown that early treatment with metformin was able to reduce central obesity and normalize circulating concentrations of insulin and adipokines, due to its positive effect on glucose, lipid, and corticosteroid metabolism.^[17]^

There are many reports on the treatment of PP with GnRH or GnRHa,^[18,19]^ but few on the treatment of LBW-PP girls with metformin. In this regard, it is of paramount importance to explore the effect of metformin on PP. This study hypothesized that the use of metformin may have a positive effect of LBW-PP girls. And the earlier it is used, the better the effect. Therefore, we evaluated the effect of metformin on girls with LBW girls with PP by using a systematic review and meta-analysis.

## 2. Materials and methods

### 2.1. Search strategy

A comprehensive literature search was conducted to identify all potentially relevant articles by using the PUBMED, Web of Science, and EMBASE from their inception to June 2021. All search methods were based on a systematic approach. This is consistent with the Preferred Reporting Items for Systematic Review and Meta-Analysis Protocols (PRISMA-P).

Searches for terms PP OR low birth weight girls with PP AND metformin were performed.

The involved articles in this study were from public databases (https://pubmed.ncbi.nlm.nih.gov/), and ethical approval was waived or not necessary.

This study was conducted in accordance with the guidelines of the Declaration of Helsinki. (It is a statement of ethical principles for medical research involving human beings, including research on material and data identifiable in human beings).

### 2.2. Selection criteria

The literature search was performed by 2 reviewers, all potentially eligible studies for inclusion and assessment and the data have been independently extracted. Differences were also resolved by consulting a third reviewer as required. The present study obtained additional data, if necessary, by getting in touch with the authors of the original studies.

The main inclusion criteria are as follows:

a. Randomized controlled clinical trials. Girls were randomly assigned to receive metformin or untreated.
b. Girls in the control and treatment group should meet the following conditions: age 7 to 13 years; body mass index (BMI) 18 to 22 kg/m^2^ or BMI *Z* score 0.9 to 1.8; testosterone ≥25 ng/dL; (bone mineral density [BMD] 0.7–0.8 g/cm^2^. In addition, in all girls, PP was attributed to exaggerate adrenarche, based on high serum androstenedione and/or dehydroepiandrosterone-sulfate (DHEAS) levels. All the variables studied (such as BMD, testosterone, androstenedione, BMI, DHEAS, sex hormone-binding globulin (SHBG), low-density lipoprotein (LDL) cholesterol, high-density lipoprotein (HDL) cholesterol, fat mass, lean mass, and gaseous insulin) were adequately described and measured in the included studies.
c. Studies reported standard mean difference (SMD) or weighted mean difference (WMD) with corresponding 95% confidence intervals (CIs) provided adequate data to calculate these values.

### 2.3. Data extraction

This form was made by 2 researchers used to extract data from the eligible studies independently. Discussions around these data were conducted to resolve discrepancies. The collected information included the author, age at study start, year of publication, sample size, treatment method, weighted mean difference (WMD; 95% CI) or SMD (95% CI), and variables controlled for matching or multivariable models. The data were inputted into the Review Manager software (RevMan 5.3). The Cochrane score was used to evaluate the quality of the selected literature as per the quality standards of the Cochrane scale. Two reviewers resolved their differences through discussion. Differences could be resolved by consulting a third reviewer when necessary.

### 2.4. Data analysis

According to each study, 12 variables were extracted as mean ± standard deviation (SD). Some researches provided 95% CI of means and standard error of means (SEM), and then 95% CIs and SEM were converted to SD values. Our study used Stata (version 12.0) to analyze data. *P* values were 2-sided, and *P* < .05 was considered as the limit of statistical significance. Six studies were used to estimate the heterogeneity. According to the heterogeneity standard of *I*² statistic, the *I*² statistic was used to assess the heterogeneity between studies. As *I*² ≥ 50%, heterogeneity was considered significant. Thus, random effects estimates were performed to calculate.^[20]^ If *I*² < 50%, fixed effects model was performed. The WMD for continuous variables were used to explain results with the 95% CI. In addition, in-depth research on subgroup (or regression) analysis and sensitivity analysis were conducted against some data with significant heterogeneity.

## 3. Results

### 3.1. Literature search and study characteristics

**Figure 1.**
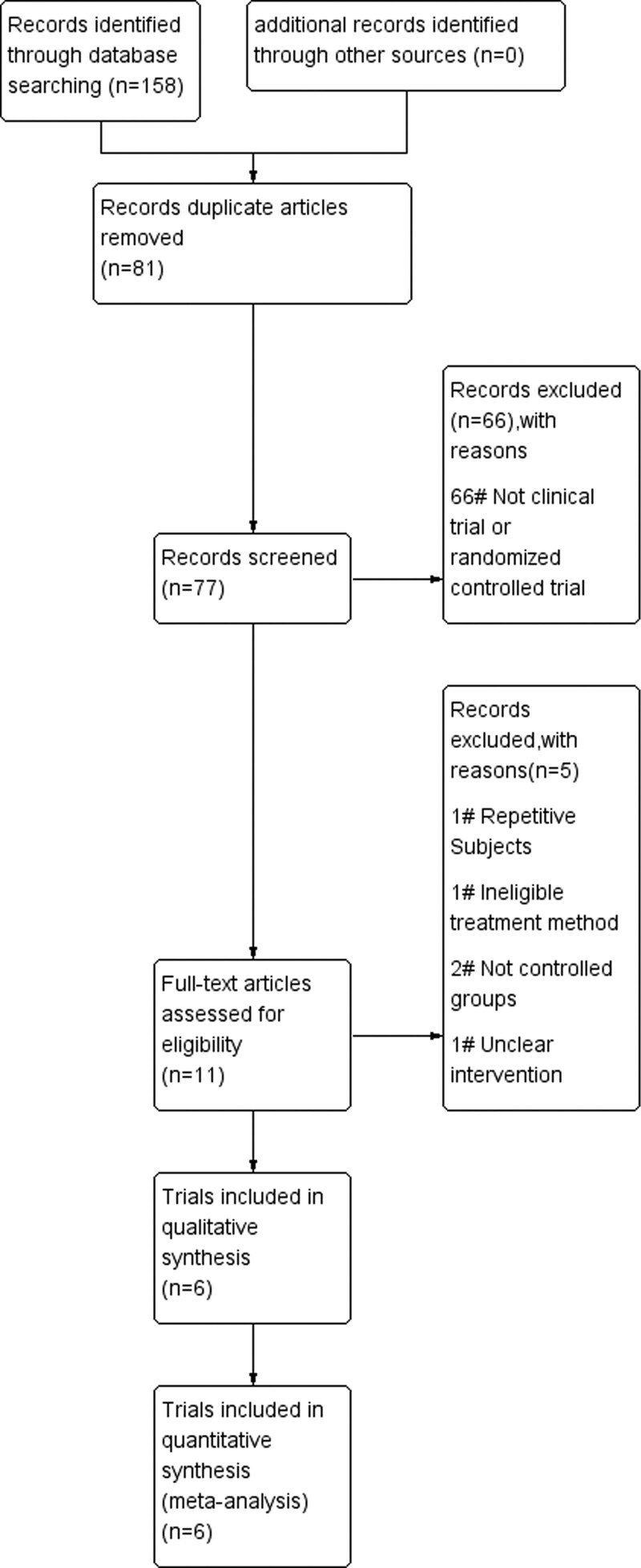
Flow diagram of study selection.

The research selection process is summarized in Figure 1. The 158 unique references were retrieved on literature searching, of which 81 were considered duplicate, and 66 of these articles were excluded due to inappropriate article type. Of the remaining 11, 5 have been excluded due to sample characteristics (e.g., ineligible treatment method) or lack of relevant data (e.g., unclear intervention). Therefore, a total of 6 researches were eligible for data extraction and were included in the meta-analysis. The characteristics of these are summarized in Table 1.

**Table 1:** The characteristics of included studies in this meta-analysis.

Study identifier	Year	Study topic	Sample (N)		Age at study start (yr)	Treatment method	Significant results
			T	U			
Ibáñez et al^[21]^	2004	Insulin sensitization early after menarche prevents progression from precocious pubarche to polycystic ovary syndrome	12	12	12.4 ± 0.2	Receive metformin (850 mg/d) for 12 mo	①②④⑥⑦⑧⑨⑩⑪
Ibáñez et al^[22]^	2004	Insulin sensitization for girls with precocious pubarche and with risk for polycystic ovary syndrome: effects of prepubertal initiation and postpubertal discontinuation of metformin treatment	16	17	8.0 ± 0.1	Receive metformin (425 mg/d) once daily at dinner time for 6 mo	①②③④⑥⑦⑧⑨⑩⑪⑫
Ibáñez et al^[23]^	2008	Metformin treatment for 4 yr to reduce total and visceral fat in low birth weight girls with precocious pubarche	19	19	7.9 ± 0.1	Receive metformin 425 mg for 2 yr, then 850 mg for 2 yr	①③④⑤⑥⑦⑧⑨⑩⑪⑫
Ibáñez et al^[24]^	2010	Pubertal metformin therapy to reduce total, visceral, and hepatic adiposity	19	19	7.9 ± 0.1	Receive metformin for 4 yr, once daily at dinner time (425 mg for 2 yr, then 850 mg for 2 yr)subsequently, all girls were monitored for 1 yr without intervention	①②③④⑤⑥⑦⑧⑨⑩⑪⑫
Ibáñez et al^[25]^	2011	Early metformin therapy to delay menarche and augment height in girls with precocious pubarche	19	19	8 ± 0.2	Receive metformin for 4 yr, once daily at dinner time (425 mg for 2 yr, then 850 mg for 2 yr)	①②③④⑤⑥⑦⑧⑨⑩⑪
de Zegher et al^[26]^	2018	Metformin for rapidly maturing girls with central adiposity: less liver fat and slower bone maturation	17	17	8 ± 0.2	Receive metformin for 4 yr, once daily at dinner time (425 mg for 2 yr, then 850 mg for 2 yr)	③⑤⑪

Values are mean ± SD. ①Testosterone; ②Androstenedione; ③BMD; ④Triglycerides; ⑤BMI Z-score; ⑥DHEAS; ⑦SHBG; ⑧LDL cholesterol; ⑨HDL cholesterol; ⑩Fat mass; ⑪Lean mass; ⑫Fasting insulin.

BMD = bone mineral density, BMI = body mass index, DHEAS = dehydroepiandrosterone-sulfate, HDL = high-density lipoprotein, LDL = low-density lipoprotein, SD = standard deviation, SHBG = sex hormone-binding globulin, T = trentment group, U = untrented group.

### 3.2. Risk of bias for all studies

**Figure 2.**
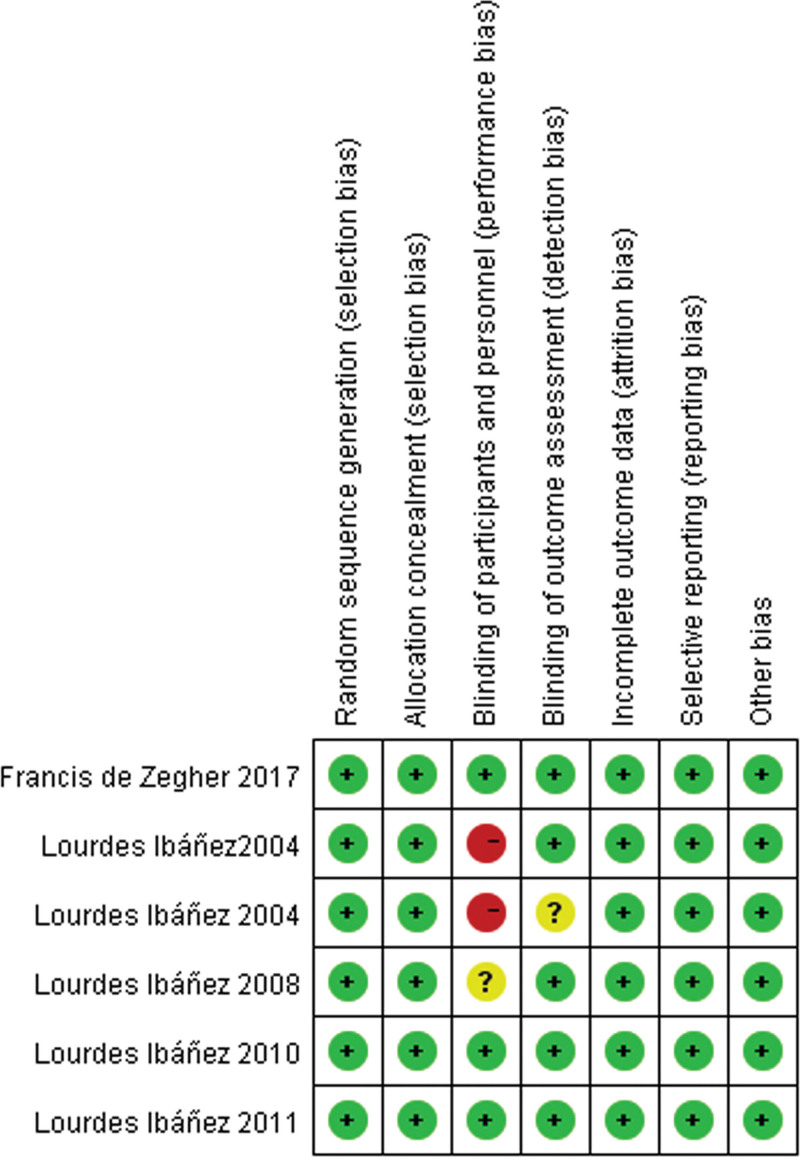
Summary of risk for each included study.

For each selected randomized, prospective and non-randomized clinical study, the risk of bias was assessed as per the standards described in the Cochrane Reviewers Handbook.^[27]^ The precise of bias risk for each study included is shown in Figure 2. In addition, Table 2 shows oxford quality scoring system (The Jadad scale).

**Table 2:** Oxford quality scoring system (The Jadad scale).

Article	Scoring items	Score
Article 1 (de Zegher et al^[21]^)	Random sequence production	2
	Allocation concealment	2
	Binding method	2
	Withdrawal	1
	Jadad Score	7
Article 2 (Ibáñez et al^[22]^^,[23]^)	Random sequence production	2
	Allocation concealment	1
	Binding method	2
	Withdrawal	1
	Jadad Score	6
Article 3 (Ibáñez et al^[22]^^,[23]^)	Random sequence production	2
	Allocation concealment	1
	Binding method	1
	Withdrawal	1
	Jadad Score	5
Article 4 (Ibáñez et al^[24]^)	Random sequence production	2
	Allocation concealment	2
	Binding method	1
	Withdrawal	1
	Jadad Score	6
Article 5 (Ibáñez et al^[25]^)	Random sequence production	2
	Allocation concealment	2
	Binding method	2
	Withdrawal	1
	Jadad Score	7
Article 6 (Ibáñez et al^[26]^)	Random sequence production	2
	Allocation concealment	2
	Binding method	2
	Withdrawal	1
	Jadad Score	7

### 3.3. Effect of metformin on testosterone

**Figure 3.**
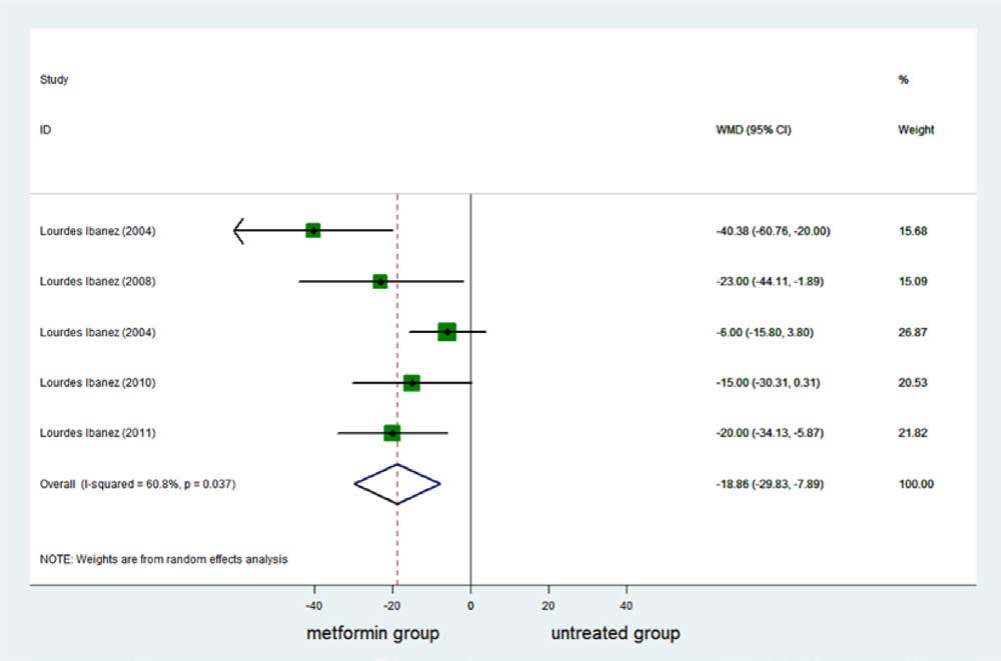
Forest plot of meta-analysis of the association about testosterone. The reduction of testosterone was higher significantly in metformin group than in control group. CI = confidence interval, WMD = weighted mean difference.

Testosterone was reported in 5 studies. A complete of 171 girls were included, of whom 85 had been in the metformin group and 86 had been in the untreated group. Testosterone decreased significantly in the metformin group (Table 3; Fig. 3).

**Table 3:** Summary of meta-analysis results.

Outcome	No. of studies	No. of participants	Type of meta-analysis	Effect estimate (95% CI)	WMD	*P* value	*I*^2^ (%)	Egger test (*P* value)
Testosterone	5	171	WMD (random)	−29.830 to −7.891	−18.86	.001	60.8	.04
Androstenedione	4	133	WMD (random)	−91.649 to −7.170	−49.41	.022	72.8	.085
BMD	5	181	WMD (fixed)	−0.008 to 0.050	0.02	.151	0.0	.793
Triglycerides	5	171	WMD (fixed)	−36.430 to −17.894	−27.16	≤.001	5.8	.002
BMI Z score	4	148	WMD (fixed)	−1.365 to −0.385	−0.87	≤.001	0.0	.005
DHEAS	5	171	WMD (fixed)	−34.579 to 0.222	−17.18	.053	0.0	.332
SHBG	5	171	WMD (fixed)	0.000 to 0.267	0.13	.049	24.9	.262
LDL cholesterol	5	171	WMD (random)	−23.044 to −1.878	−12.46	.021	53.5	.429
HDL cholesterol	5	171	WMD (fixed)	3.707 to 9.826	6.76	≤.001	0.0	.372
Fat mass	5	171	WMD (fixed)	−4.983 to −1.971	−3.48	≤.001	0.0	.661
Lean mass	6	205	WMD (fixed)	0.158 to 2.354	1.26	.025	0.0	.772
Fasting insulin	3	114	WMD (fixed)	−5.454 to −1.215	−3.33	.002	0.0	.048

BMD = bone mineral density, BMI = body mass index, CI = confidence interval, DHEAS = dehydroepiandrosterone-sulfate, HDL = high-density lipoprotein, LDL = low-density lipoprotein, SHBG = sex hormone-binding globulin.

### 3.4. Effect of metformin on androstenedione

**Figure 4.**
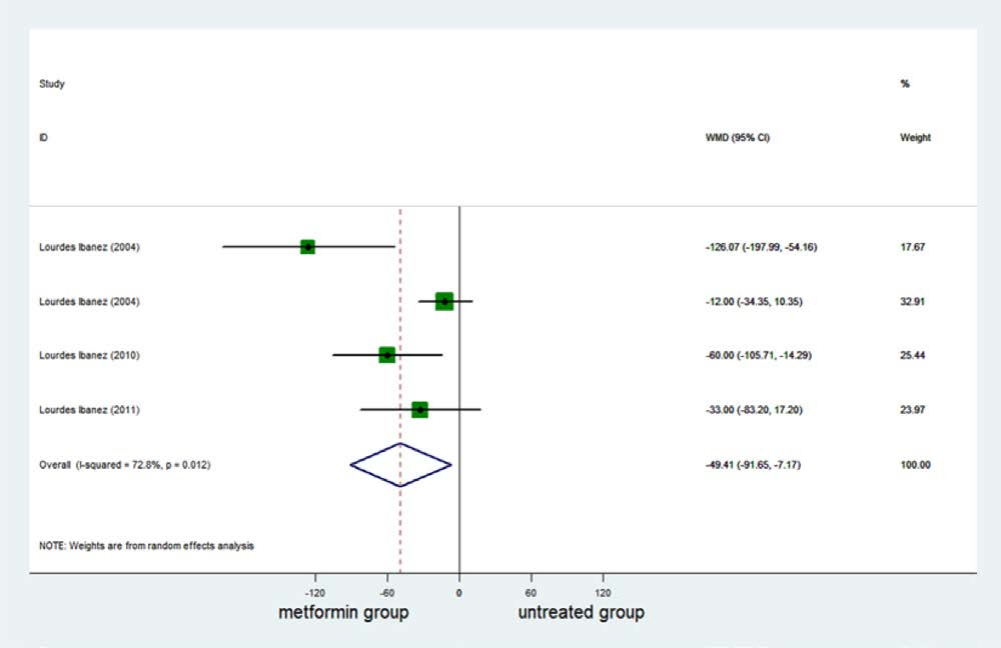
Forest plot of meta-analysis of the association about androstenedione. The reduction of androstenedione was higher significantly in metformin group than in control group. CI = confidence interval, WMD = weighted mean difference.

Androstenedione was reported in 4 studies. A complete of 133 girls were included, of whom 66 had been in the metformin group and 67 had been in the untreated group. Androstenedione decreased significantly in the metformin group (Table 3; Fig. 4).

### 3.5. Effect of metformin on BMD

**Figure 5.**
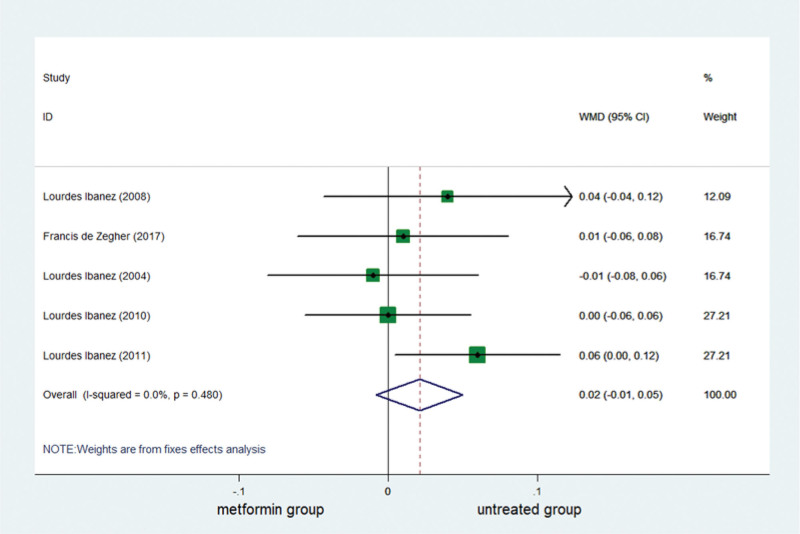
Forest plot of meta-analysis of the association about BMD. No significant distinction between the metformin and untreated groups in terms of BMD. BMD = bone mineral density, CI = confidence interval, WMD = weighted mean difference.

BMD has been reported in 5 studies. A complete of 181 girls were included, of whom 90 had been in the metformin group and 91 had been in the untreated group. The combined results confirmed no significant difference in BMD between the metformin and untreated groups (Table 3; Fig. 5).

### 3.6. Effect of metformin on BMI *Z* score

**Figure 6.**
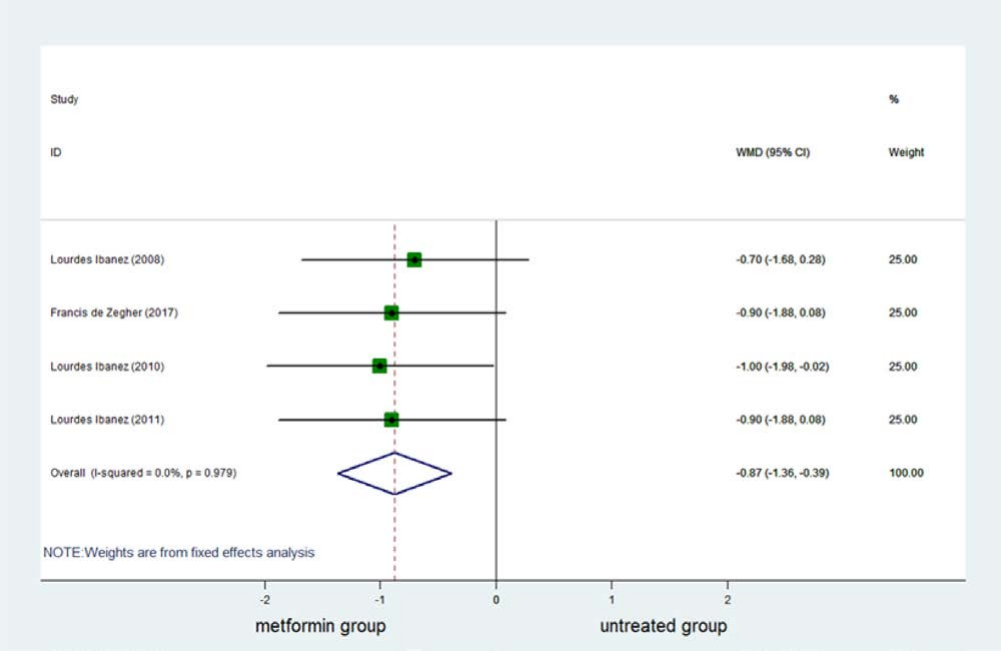
Forest plot of meta-analysis of the association about BMI *Z* score. The reduction of BMI *Z* score was higher significantly in metformin group than in control group. BMI = body mass index, CI = confidence interval, WMD = weighted mean difference.

BMI *Z* score was reported in 5 studies. A complete of 148 girls were included, of whom 74 had been in the metformin group and 74 had been in the untreated group. BMI *Z* score decreased significantly in the metformin group (Table 3; Fig. 6).

### 3.7. Other outcomes

Table 3 shows a summary of the meta-analysis outcomes, which included testosterone, androstenedione, BMD, triglycerides, BMI *Z* score, DHEAS, SHBG, LDL cholesterol, HDL cholesterol, fat mass, lean mass, and fasting insulin. In terms of lipid metabolism, the triglycerides, LDL cholesterol, and HDL cholesterol of the treatment group had a positive effect when compared with the control group. In addition, insulin resistance and obesity were improved. It was found that fasting insulin, fat mass, and BMI *Z* score in the treatment group were significantly referenced than those in the untreated group but lean mass increased.

**Figure 7.**
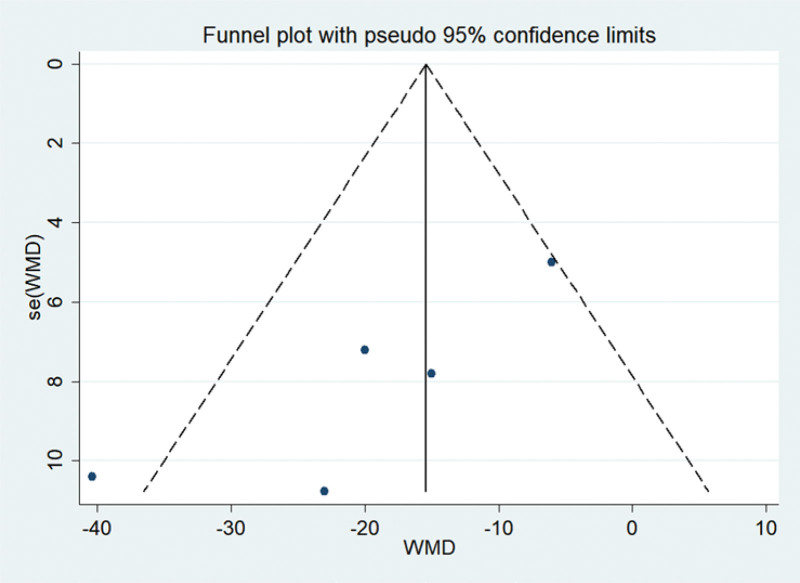
Funnel plots of publication bias. WMD = weighted mean difference.

The results of publication bias are shown in Table 3. Figure 7 shows Begg funnel plots estimating publication bias.

## 4. Discussion

**Figure 8.**
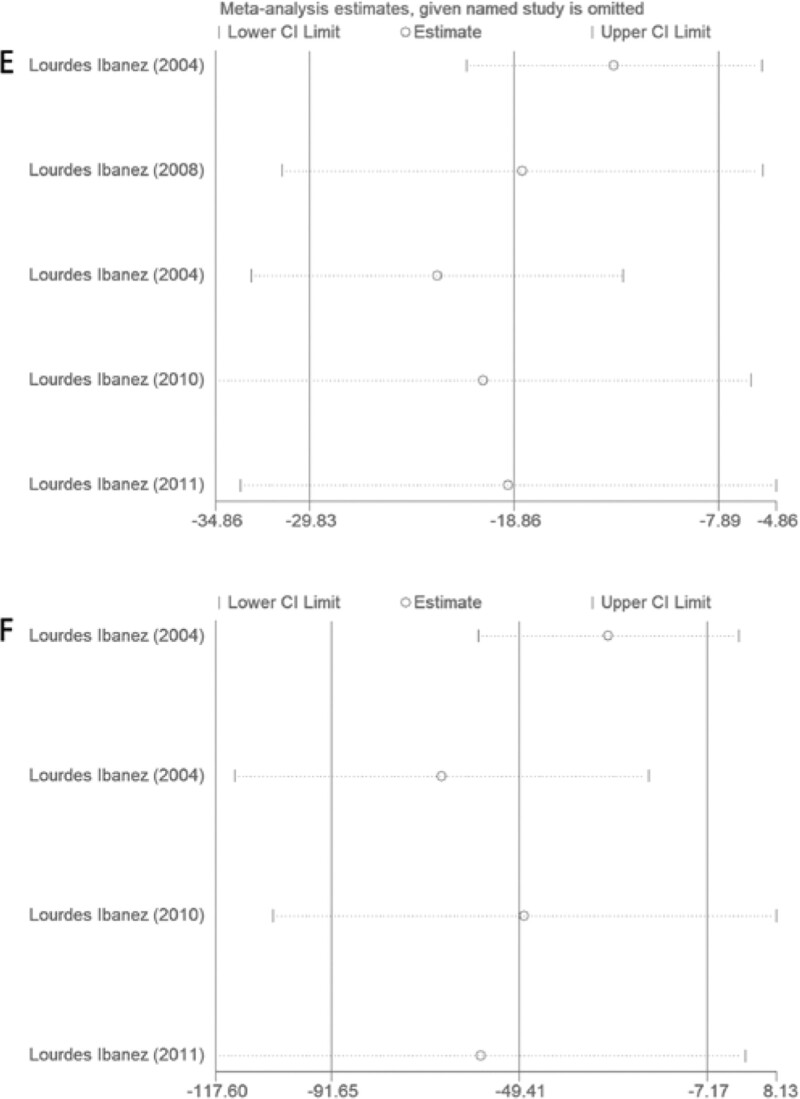
Sensitivity analysis to evaluate E(testosterone) and F(androstenedione). CI = confidence interval.

The main objective of this study is to assess the effect of metformin on LBW-PP girls. Through the Jadad scale, we evaluated all the articles included, all of which were of high quality. According to meta-analysis, the levels of testosterone and androstenedione in the metformin intervention group were significantly lower than those in the untreated group. It is possibly attributable that metformin inhibits testosterone-induced endoplasmic reticulum stress in ovarian granulosa cells via inactivation of p38 mitogen-activated protein kinases (MAPK).^[28]^ The mechanism by which metformin reduces testosterone levels in girls might be related to the improvement of peripheral insulin resistance.^[29]^ In addition, high heterogeneity was detected, which might be due to different ages and administration methods. However, several subgroup analyses (for dosing regimens and age groups) include few studies. Therefore, the results provided by subgroup analysis are not as reliable as the whole, and sensitivity analysis shows that the studies Ibáñez et al^[21,22]^ have significant sensitivity (Fig. 8). Based on the data of testosterone, the baseline of the older age group is relatively high. Therefore, the value changes significantly after the intervention. In addition, the length of the medication may render significant different effects. A small amount of short-term medication and its lowering effect are not as effective as adequate long-term medication, which is consistent with the perspective of some associated research reports.^[30]^

The data showed that there was no significant difference in DHEAS between the metformin group and the untreated group. The results of the present study does not support the evidence that metformin can reduce DHEAS levels. Nonetheless, some researches have shown that metformin has a certain regulatory effect on DHEAS. Metformin can regulate the sensitivity of insulin levels.^[31]^ Besides, metformin can improve the oxidative stress status of girls to reduce the level of androgen and DHEAS.^[17,32]^ Due to the limitation of the number of included articles, further verification is required for the present research results.

Associated research reports showed that metformin has little effect on DHEAS in girls with PCOS of childbearing age.^[33]^ Hence, early medication can adjust over-excited adrenal function and reduce the level of DHEAS. PCOS girls have missed this window period. As human body develops, it is difficult to get fundamental improvement after the window period is missed.

In addition, the data showed that the levels of SHBG in the metformin intervention group were significantly lower than those in the untreated group. Based on the results above, the effect of metformin on testosterone, androstenedione, and SHBG can be judged: metformin has a positive effect on reducing the risk of LBW-PP girls developing into PCOS.^[34–37]^ By reducing insulin resistance in the liver, decreasing androgen secretion by adrenal glands and ovaries, and increasing the production of SHBG in the liver, metformin takes effect in girls, thus reducing free testosterone concentrations.^[38–40]^

The results of this meta-analysis show that there was no significant difference in BMD between metformin group and untreated group, which is consistent with the conclusions in the study by de Zegher et al.^[26]^ The effect of metformin on the bone development of LBW-PP girls requires further studies. Our meta-analysis also showed the metformin-treated group presented consistent improvements in all biochemical and body composition variables when compared to the untreated group. According to the results in this present study, LDL cholesterol, BMI *Z* score, fat mass, and fasting insulin significantly reduced. HDL cholesterol and lean mass significantly increased, indicating that metformin can effectively reduce the risk of LBW girls with PP women developing obesity and hyperlipidemia. In addition, it has been found that lean mass in the metformin intervention group increased significantly when compared to the untreated group. This contradicted the conclusion that metformin had no effect on lean mass in the present study.^[26]^ However, the sample size is small, which is not sufficient to draw a definite conclusion.

The result of meta-analysis showed that early and adequate use of metformin has a more significant effect on girls with LBW-PP girls. Its mechanism may be as follows: The adolescence provides an opportunity to reprogram the wrong programming that occurred in early life. For PP girls, a catch-up growth after birth showed up, thus resulting in the wrong coding of early life planning. Adolescence may be a critical window in which the epigenetic settings may be changed.^[25]^ Using metformin at this phase can reduce the risk of obesity and hyperlipidemia in LBW-PP girls more effectively, regulate the secretion of androgens in the adrenal glands, and reduce the future the risk of developing into PCOS. Regarding the timing of medication, it may be demarcated by menarche, which requires further study.

### 4.1. Limitations and suggestions

Some limitations should be considered before the results of this meta-analysis were examined. The number of girls included in each group of randomized controlled trial (RCT) is relatively small. In addition, the dose of metformin ranged from 425 to 825 mg in different studies and the treatment time varied from as short as 6 months to as long as 5 years. In addition, significant heterogeneity in eligible studies adversely affected the meaningful results of the current meta-analysis. Besides, the very low quality for testosterone, triglycerides, BMI *Z* score, and fasting insulin was the result of high probability of publication bias, as indicated by their Egger test *P* values of .040, .002, .005, and .048. Although this present study has certain limitations, results have shown that metformin can have positive effects. It is suggested that more scientifically designed, randomized, double-blind controlled clinical trials are required to support the results of this research.

## 5. Conclusion

This evidence of the efficacy of metformin in girls with low birth weight PP has been provided in meta-analysis, which proved that metformin was able to boost metabolism and weight loss. Metformin has been proven positive in preventing low birth weight precocious puberty from developing into obesity and polycystic ovarian syndrome.

## Author contributions

ZL and XS conceived and designed the study; ZL and YW searched the related articles; ZL, XS, YW, and LL analyzed the data; ZL, XS, and LL wrote the manuscript. JZ supervised the whole process. All authors read and approved the final manuscript.
